# Viral Genomic Footprints in Breast Cancer: A Systematic Review and Meta-Analysis of Tissue-Based Detection of Epstein–Barr Virus and Bovine Leukemia Virus

**DOI:** 10.3390/ijms27104452

**Published:** 2026-05-15

**Authors:** Georgia Margioula-Siarkou, Chrysoula Margioula-Siarkou, Eleftherios Vavoulidis, Stefanos Flindris, Stamatios Petousis, Costas Haitoglou, Georgios Mavromatidis, Konstantinos Dinas

**Affiliations:** 1 2nd Department of Obstetrics and Gynaecology, Aristotle University of Thessaloniki, 54642 Thessaloniki, Greece; 2 Laboratory of Biochemistry, School of Medicine, Aristotle University of Thessaloniki, 54124 Thessaloniki, Greece; 3 3rd Department of Obstetrics and Gynaecology, Aristotle University of Thessaloniki, 54642 Thessaloniki, Greece

**Keywords:** breast cancer, viral carcinogenesis, Epstein–Barr virus, Bovine Leukemia virus, molecular detection

## Abstract

Viral carcinogenesis as a causative mechanism of breast cancer has been intensively researched during the last decades. The role of Epstein–Barr virus (EBV) and Bovine Leukemia virus (BLV) in breast oncogenesis has been investigated in a plethora of studies, but with conflicting results. The aim of this systematic review and meta-analysis was to compare the frequency of molecular detection of the EBV genome and BLV genome between women with breast cancer and women without malignant breast tumors. This systematic review and meta-analysis adhered to the Preferred Reporting Items for Systematic Reviews and Meta-Analyses (PRISMA) guidelines. MEDLINE, SCOPUS, Cochrane CENTRAL and ClinicalTrials.gov were searched up to 20 May 2024. Included studies were those comparing the frequency of molecular detection of the EBV and/or BLV genome in breast tissue specimens with polymerase chain reaction (PCR) methods between patients with breast cancer and women without breast malignancies. The primary outcomes of the study were the frequency of molecular detection of the EBV genome and BLV genome. Methodological quality of included studies was assessed using the Newcastle–Ottawa Scale. A total of 29 studies met the selection criteria and were included in this meta-analysis; 19 studies reported results for molecular detection of the EBV genome, 9 studies for detection of the BLV genome and 1 study for detection of genomic material of both viruses. The frequency of molecular detection of viral genomes was significantly higher in patients with breast cancer, compared to women with healthy breasts or benign breast diseases, regarding both EBV (OR: 3.041, 95% CI: 1.791 to 5.164, *p* < 0.0001) and BLV (OR: 3.459, 95% CI: 2.118 to 5.650, *p* < 0.0001). The frequency of molecular detection of EBV and BLV genomes is higher, in a statistically significant manner, in patients with breast cancer compared to women without breast malignancies. The presence of these viral factors in breast tissue could imply their potential contribution in breast carcinogenesis, but is not sufficient to establish it, and the molecular detection of their genomes could be potentially exploited in the future for preventive, diagnostic and therapeutic purposes. Further studies are required to thoroughly investigate and establish a causal relationship between EBV and BLV infection and breast carcinogenesis, as well as to support the use of viral genome molecular detection in clinical settings for the management of breast cancer patients.

## 1. Introduction

Breast cancer represents the leading cancer diagnosis among women globally, accounting for a substantial proportion of female malignancies [1]. The rapid progress during recent decades in the fields of molecular biology and genetics has allowed a significant expansion of our knowledge regarding the pathophysiology of breast carcinogenesis and, with the exception of cases with clear and known genetic predisposition, available evidence indicates that breast carcinogenesis results from the complex interplay of genetic susceptibility, immune regulation, environmental exposures, hormonal influences, and lifestyle-related factors [2,3,4]. However, despite intensive research efforts, the precise mechanisms driving breast carcinogenesis are still not completely clarified. In this context, viral infection has emerged as a potential contributing factor in breast cancer pathogenesis [5]. The main viral agents that have been identified in malignant breast tumors and studied as potential contributors to breast carcinogenesis, according to international literature, are human papilloma virus (HPV), Epstein–Barr virus (EBV), Bovine Leukemia virus (BLV) and Mouse Mammary Tumor Virus (MMTV) [6,7,8].

Epstein–Barr virus, a DNA herpesvirus primarily targeting B lymphocytes, estimated to have infected more than 95% of the global population, has already been linked to the pathogenesis of nasopharyngeal carcinoma, Burkitt lymphoma, and gastric cancer [9,10,11,12]. The mechanism that EBV employs to infect mammary epithelial cells remains unclear [13], although a potential role of the CD21 receptor has been suggested [14,15]. The predominant molecular pathway through which EBV may contribute to breast carcinogenesis is the activation of the c-Met signaling cascade, mediated by the viral latent membrane protein 1 (LMP-1) [14]. Bovine Leukemia virus, a retrovirus closely associated with Human T-Lymphotropic Virus (HTLV) types 1 and 2 [8], is mainly transmitted to the human population through consumption of non-pasteurized dairy products from infected cattle [16], while it is proposed that it is implicated in breast carcinogenesis by inhibiting DNA repair mechanisms in mammary epithelial cells [17,18]. The role of EBV and BLV infection in the pathophysiology of breast cancer has been previously investigated in a variety of studies, though often with contradictory findings.

### Objective

The present systematic review and meta-analysis aims to compile available data from studies published in the international literature regarding the frequency of molecular detection of EBV and BLV genomes in patients with breast cancer and women without malignant breast tumors, grouping their results by viral strain, in an effort to further investigate and elucidate the potential contribution of EBV and BLV infection to breast carcinogenesis.

The main objective of the present systematic review and meta-analysis is to compare the frequency of molecular detection of the EBV genome and BLV genome between patients with breast cancer and women without breast malignancies.

## 2. Methods

The present systematic review and meta-analysis was performed in accordance with the Preferred Reporting Items for Systematic Reviews and Meta-Analyses (PRISMA) guidelines [19] (Supplementary File S1).

### 2.1. Eligibility and Inclusion Criteria

Studies eligible for inclusion were those reporting quantitative data on molecular detection of EBV and/or BLV genomic material in patients with breast cancer and in women without breast malignancies. Regarding the study population, eligible studies included patients of any race and age with histologically confirmed breast cancer, either invasive or in situ, of any histological subtype or disease stage, as well as healthy women without known breast disease or patients with histologically confirmed benign breast diseases. Eligible studies also used polymerase chain reaction (PCR)-based molecular techniques for detection of viral genomes (real-time PCR, nested PCR, in situ PCR, liquid-phase PCR), performed on breast tissue specimens obtained either via core needle biopsy, open surgical biopsy or after definitive surgical treatment. As for study types, observational studies, prospective or retrospective cohort studies, case–control studies, and cross-sectional studies were considered eligible.

Studies were excluded if they performed molecular detection of the EBV and/or BLV genome in male subjects or animal models. Case–control studies using dependent samples, namely those in which control samples consisted of serum or histologically normal breast tissue collected from the peripheral margins of malignant breast tumors from patients with breast cancer, without an independent control population, were considered ineligible. Additionally, case–control studies where the case group did not exclusively consist of patients with invasive or in situ breast malignancies, or the control group included patients with premalignant breast lesions, were also excluded. Furthermore, single-arm studies reporting molecular detection exclusively in patients with breast cancer, without comparison to control groups, as well as studies with unclear or insufficient numerical data, were excluded from the meta-analysis. Studies employing solely laboratory methods for viral detection other than PCR, such as immunohistochemistry, in situ hybridization, mass spectrometry, enzyme-linked immunosorbent assay (ELISA), serological antibody detection assays, or using biological material other than breast tissue specimens were also excluded. Likewise, studies investigating molecular detection of viral genomes other than EBV or BLV were not considered eligible. Finally, case reports or case series, narrative reviews, systematic reviews and meta-analyses, comments, letters to the editor, retracted articles, and studies published in languages other than Greek, English, or French were excluded from the present study.

### 2.2. Types of Outcome Measures

The primary outcome measures of the present and meta-analysis were to compare the frequency of EBV genome and BLV genome molecular detections independently, as well as simultaneous detections of EBV and BLV genomes in breast tissue specimens from patients with breast cancer and from healthy women or patients with benign breast diseases.

### 2.3. Information Sources and Search Strategy

A comprehensive literature search was conducted in the MEDLINE, SCOPUS, and Cochrane Central Register of Controlled Trials (CENTRAL) databases, as well as in the clinical trial registry ClinicalTrials.gov, up to 20 May 2024. The search strategy employed a combination of the following keywords: “breast cancer,” “breast carcinoma,” “breast tumour,” “breast tumor,” “breast neoplasm,” “Epstein-Barr,” “Epstein Barr,” “EBV,” “bovine leukemia virus,” and “BLV” (Supplementary File S2). Truncation symbols were additionally applied to keywords in order to broaden the search results and minimize the risk of omitting potentially eligible studies. A secondary literature search was subsequently performed by perusing the references of retrieved articles, as well as the included studies of previously published meta-analyses addressing the same research topic.

### 2.4. Study Selection and Data Extraction

All records identified through the database searches were imported into a reference management software (Mendeley Desktop Version 1.19.4) for further evaluation. Following removal of duplicate records using the software’s dedicated tool, all search results were independently screened by two of the authors (G.M.-S. and S.P.). After initial screening based on title and abstract, a full-text review of all relevant remaining studies was undertaken. Any disagreements during study selection were resolved through discussion or consultation with a third reviewer (C.M.-S.). Data extraction from included studies was conducted using a predefined electronic form to record various studies and patient characteristics. In studies where molecular detection of EBV and/or BLV genomes was performed in additional patient groups or biological materials, data extraction was limited to the information relevant to the outcomes of the meta-analysis. In case of missing data, corresponding authors were contacted for additional information.

### 2.5. Quality Assessment of Individual Studies

Methodological quality of the included studies was independently assessed by two authors (G.M.-S. and S.P.) using the Newcastle–Ottawa Scale, a tool recommended by Cochrane for quality assessment of non-randomized studies [20]. This scale evaluates cohort and case–control studies based on eight items, covering three methodological domains: selection of study groups, comparability between groups, and outcome assessment. Study quality is rated using a star-based system ranging from 0 to 9 stars. Consistent with the approach adopted in recent meta-analyses employing this tool [21,22], studies scoring 7–9 stars were classified as high quality, those scoring 4–6 stars were methodologically moderate and those scoring 0–3 stars were considered low quality.

### 2.6. Summary Measures and Synthesis of the Results

Since the outcomes of the meta-analysis were categorical, the odds ratio (OR) with 95% confidence intervals (95% CIs) were calculated for each outcome. Meta-analysis was conducted when sufficient data was provided from at least two studies for an outcome. Data synthesis was performed using a random-effects model (DerSimonian and Laird method), with statistical significance set at *p* < 0.05 (two-tailed *p*-value). All statistical analyses were carried out using Comprehensive Meta-Analysis software, version 3.3.070 [23].

### 2.7. Publication Bias

To investigate the presence of publication bias, it was decided a priori to construct funnel plots for outcomes gathering data from at least ten studies. Funnel plots were visually evaluated, and in case asymmetry was detected, exploratory analyses using appropriate statistical tests were planned.

## 3. Results

### 3.1. Study Selection

**Figure 1:**
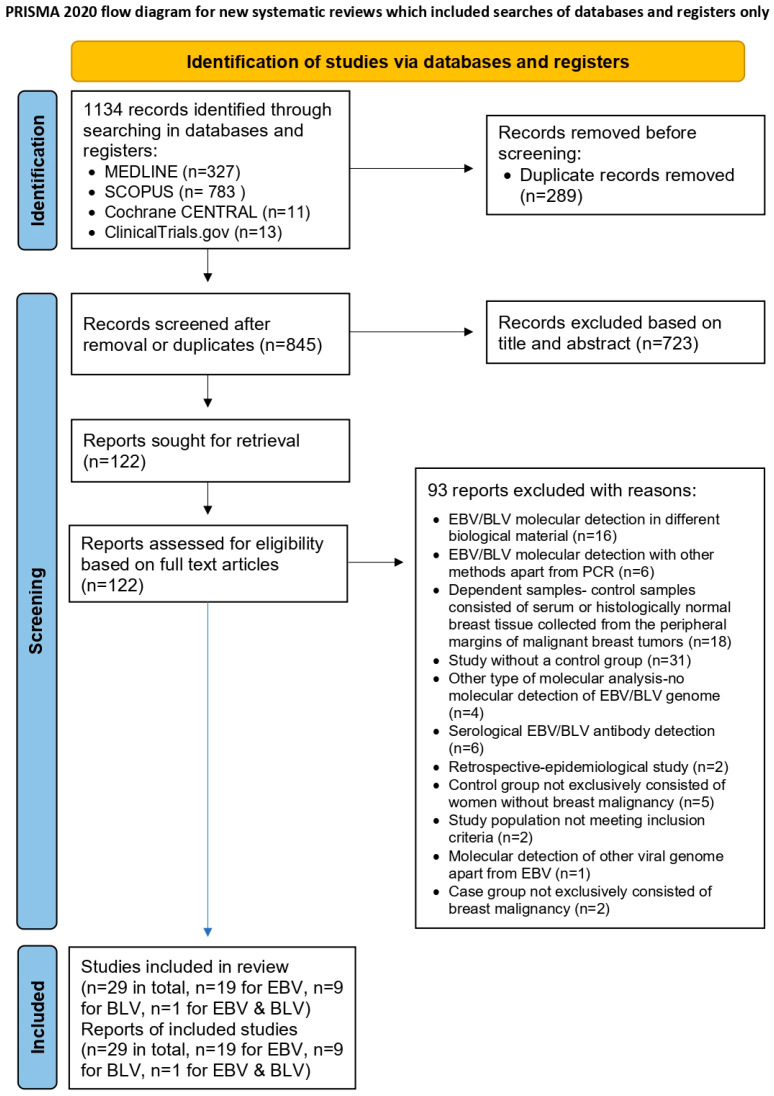
PRISMA flowchart of study selection [19].

The database search and secondary hand searching yielded a total of 1134 records for assessment. After removal of 289 duplicate records, 845 remaining studies were screened and 723 were excluded based on title and abstract, due to irrelevance or apparent non-compliance with the eligibility criteria. The full texts of the remaining 122 studies were assessed, resulting in the exclusion of 93 studies with clear justification (Supplementary File S3). Ultimately, 29 studies [8,13,16,17,24,25,26,27,28,29,30,31,32,33,34,35,36,37,38,39,40,41,42,43,44,45,46,47,48] that fulfilled all eligibility criteria, published between 2005 and 2024, all case–control studies in regard to their design, were included in the present systematic review and meta-analysis, collectively enrolling 2572 patients with histologically confirmed invasive or in situ breast carcinomas and 1467 women with either healthy breasts or histologically confirmed benign breast disease. The search strategy and study selection process are summarized in the PRISMA flowchart (Figure 1).

### 3.2. Study Characteristics and Quality Assessment of Individual Studies

The characteristics of the included studies are summarized in Table 1. With respect to viral detection, 19 studies [13,24,25,26,27,28,29,30,31,32,33,34,35,36,37,38,39,40,41] reported molecular detection of EBV genetic material, 9 studies [16,17,42,43,44,45,46,47,48] focused on BLV genome detection, and 1 study [8] provided data for detection of both EBV and BLV genomes. As for the study populations, 27 studies [8,13,16,17,24,25,26,27,28,29,30,31,32,33,34,35,36,38,40,41,42,43,44,45,46,47,48] included exclusively patients with invasive breast cancer in the case groups, while 2 studies [37,39] included both invasive and in situ breast carcinoma patients. Regarding the control groups, 14 studies [8,16,29,30,31,32,34,37,38,39,44,45,46,48] included only women with healthy breast tissue, 11 studies [17,25,26,33,35,36,40,41,42,43,47] included only patients with histologically confirmed benign breast lesions, and 4 studies [13,24,27,28] included a mix of those two populations.

The quality assessment of included studies, performed using the Newcastle–Ottawa Scale, is presented in Table 2. Of the 29 included studies, 23 were classified as moderate methodological quality [8,13,16,17,24,25,26,27,28,29,30,33,35,36,37,39,41,42,43,44,45,46,47] and 6 studies as high quality [31,32,34,38,40,48], while none were rated as low quality.

### 3.3. Results of Individual Studies

Regarding the primary outcome measures, molecular detection of the EBV genome was reported in 20 studies [8,13,24,25,26,27,28,29,30,31,32,33,34,35,36,37,38,39,40,41], while 10 studies [8,16,17,42,43,44,45,46,47,48] provided data for molecular detection of the BLV genome. The results of the individual studies for each outcome measure are presented in detail in Table 3.

### 3.4. Synthesis of Results

**Figure 2:**
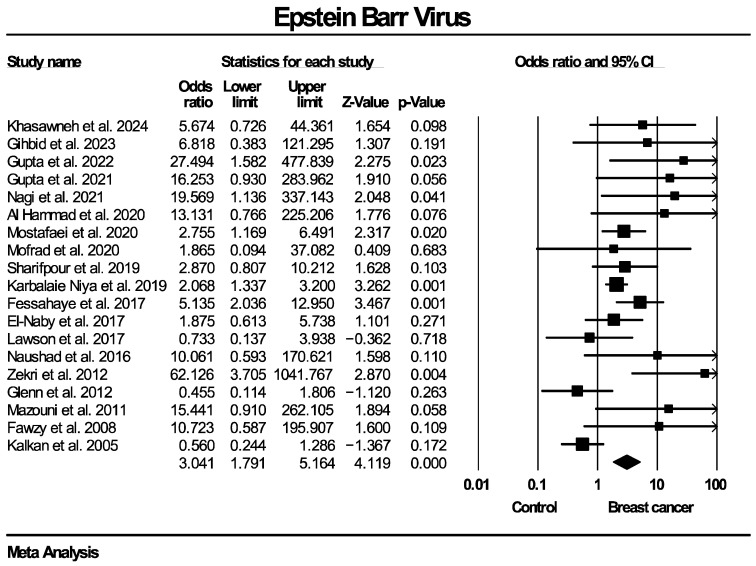
EBV molecular detection forest plot. Frequency of EBV genome molecular detection was significantly higher in patients with histologically proven breast cancer, compared to women without breast malignancies.

**Figure 3:**
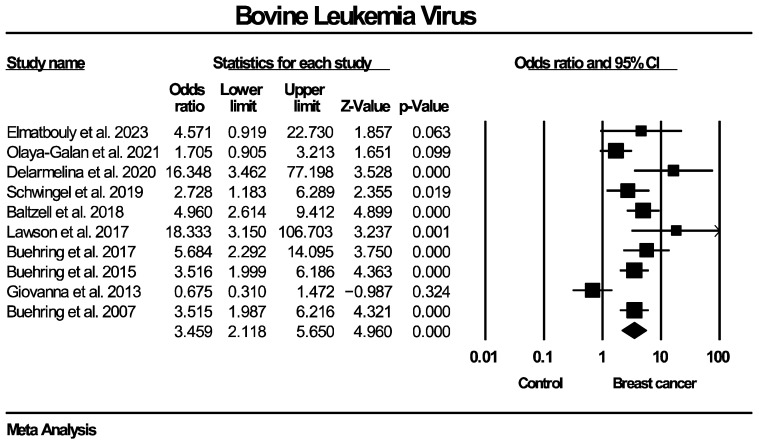
BLV molecular detection forest plot. Frequency of BLV genome molecular detection was significantly higher in patients with histologically proven breast cancer, compared to women without breast malignancies.

Twenty studies, enrolling in total 1899 patients with breast cancer and 813 women without known breast disease or with benign breast diseases, reported results on the molecular detection of the EBV genome. The frequency of EBV molecular detection was 3.041 times higher in the breast cancer group (OR: 3.041, 95% CI: 1.791–5.164, I^2^ = 55.5%) compared with the control group, and this difference was statistically significant (*p* < 0.0001). Regarding molecular detection of the BLV genome, results were provided by 10 studies, collectively enrolling 685 patients with breast cancer and 670 women without known breast disease or with benign breast diseases. The frequency of BLV molecular detection was 3.459 times higher in the breast cancer group (OR: 3.459, 95% CI: 2.118–5.650, I^2^ = 70.9%) compared with the control group, with this difference also being statistically significant (*p* < 0.0001). Forest plots for EBV and BLV molecular detection are respectively provided in Figure 2 and Figure 3. Finally, only one study provided data for simultaneous EBV and BLV genome detection in the same specimens, so it was not possible to conduct the respective meta-analysis.

**Figure 4:**
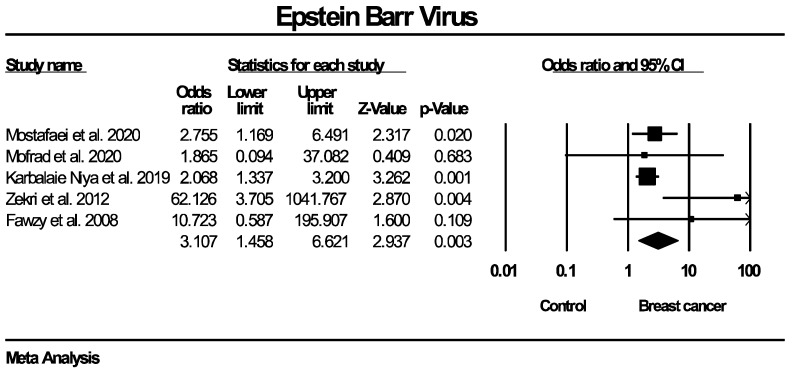
EBV molecular detection—sensitivity analysis of high-quality studies (NOS ≥ 7).

Although the overall pooled estimates were statistically significant regarding the molecular detection of both EBV and BLV, their robustness could be questioned, as the majority of included studies for both outcomes were of moderate methodological quality, according to the Newcastle–Ottawa Scale (NOS). This finding is not unexpected, since all of the included studies were observational, where limitations in comparability such as lack of adjustment for potential confounders are common. To address this concern, a sensitivity analysis restricted to high-quality studies (NOS ≥ 7) was performed separately for each outcome. For EBV genome detection, 5 out of 20 included studies were of high quality and, as shown in Figure 4; the results of the sensitivity analysis remained consistent with the primary analysis (OR: 3.107, 95% CI: 1.458–6.621, *p* = 0.003 vs. OR: 3.041, 95% CI: 1.791–5.164, *p* < 0.0001), while a reduction in heterogeneity was observed (I^2^ = 40.4% vs. 55.5%). These findings support the robustness of the reported results and suggest that study quality did not substantially influence the overall effect estimate. Regarding BLV molecular detection, only 1 study out of 10 was rated as high quality (NOS ≥ 7); therefore, a sensitivity analysis restricted to such studies was not feasible. However, the effect estimate from the single high-quality study [48] was consistent in direction with the overall pooled estimate (OR: 3.515, 95% CI: 1.987–6.216 vs. OR: 3.459, 95% CI: 2.118–5.650). Consequently, since the potential impact of study quality on the pooled estimate could not be formally evaluated, the results should be interpreted with caution.

Given the presence of moderate to substantial heterogeneity (I^2^ = 55.5% for EBV and 70.9% for BLV genome detection), additional subgroup analyses were performed to explore potential sources of variability across studies. Taking into consideration that differences regarding details of the methodological design of the included studies were identified, respective subgroup analyses were stratified according to PCR methodology (real-time PCR, nested PCR, in situ PCR), type of control group (healthy controls with normal breasts, diagnosed benign breast disease, mixed groups) and tissue preservation method (fresh-frozen versus formalin-fixed paraffin-embedded [FFPE] samples). Since BLV infection is known to be predominant mainly in North America and Asia [49], an additional subgroup analysis based on the geographical region that the population of each study originated from was also added. Each subgroup analysis was conducted separately for EBV and BLV detection. Subgroup analyses were restricted to categories with data from a minimum of two studies, in order to enable estimation of the outcome measure. Effect estimates were calculated within each subgroup using a random-effects model.

**Figure 5:**
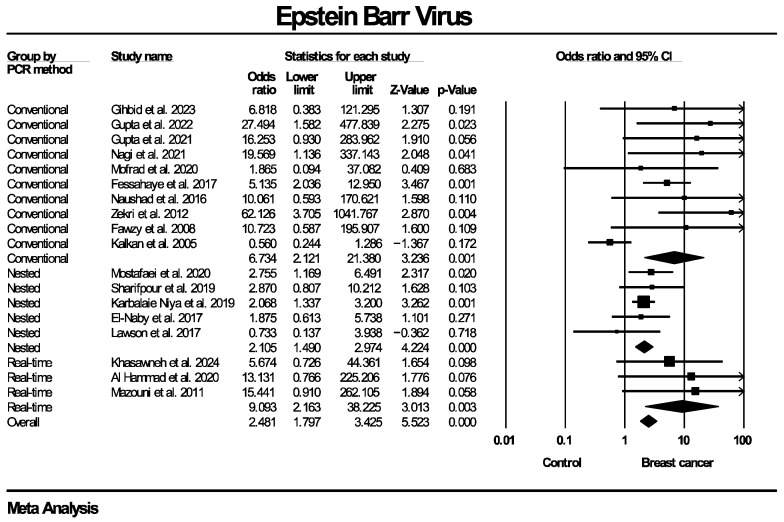
EBV molecular detection—subgroup analysis based on PCR method. Frequency of EBV genome molecular detection was statistically significantly higher in breast cancer patients compared to women without breast malignancy, across all PCR techniques employed (conventional, nested, real-time PCR).

**Figure 6:**
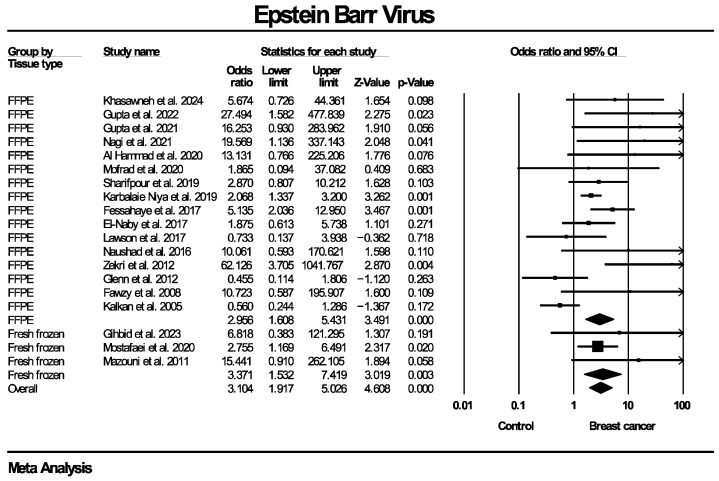
EBV molecular detection—subgroup analysis based on tissue preservation method. Frequency of EBV genome molecular detection was statistically significantly higher in breast cancer patients compared to women without breast malignancies, across all tissue preservation methods employed (fresh frozen, formalin-fixed paraffin-embedded tissue). FFPE: formalin-fixed paraffin-embedded.

**Figure 7:**
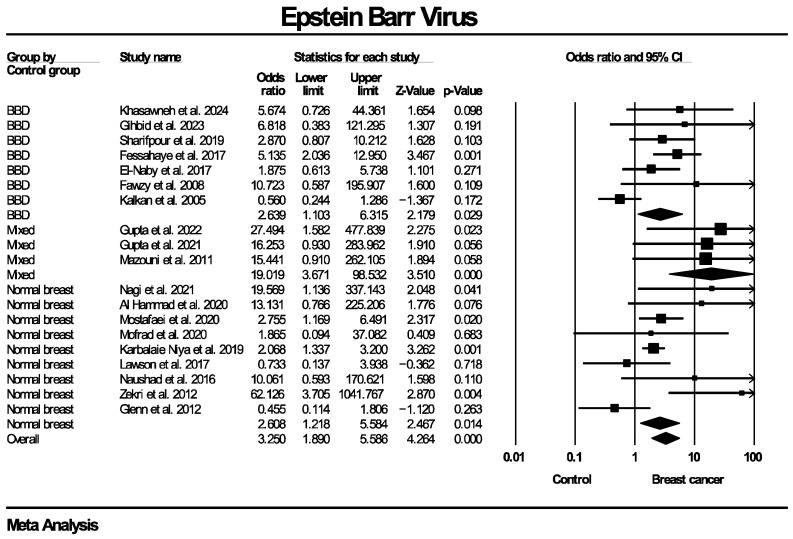
EBV molecular detection—subgroup analysis based on types of control groups. Frequency of EBV genome molecular detection was statistically significantly higher in breast cancer patients compared to women without breast malignancies, across all types of control groups used in individual studies (healthy women with normal breast, women diagnosed with benign breast diseases, mixed control groups consisting of both women with healthy breasts and benign breast diseases). BBD: benign breast disease.

**Figure 8:**
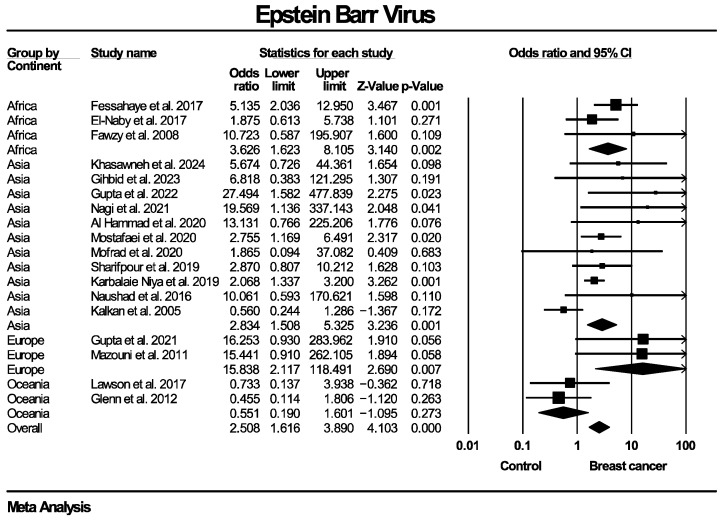
EBV molecular detection—subgroup analysis based on geographical region/continents of populations enrolled in included studies. Frequency of EBV genome molecular detection was statistically significantly higher in breast cancer patients compared to women without breast malignancies, across all evaluated geographic regions/continents, with the exception of Oceania. Note: The study by Zekri et al. enrolled women from both an African (Egypt) and an Asian country (Iraq), so it could not be delegated in a continent and was excluded from this subgroup analysis.

The corresponding forest plots for these subgroup analyses regarding EBV genome detection are presented in Figure 5, Figure 6, Figure 7 and Figure 8. As shown in the forest plots, the frequency of EBV molecular detection was consistently and significantly higher in breast cancer patients compared to controls across all PCR methods, namely conventional PCR (OR: 6.743, 95% CI: 2.121–21.380, *p* = 0.001), nested PCR (OR: 2.105, 95% CI: 1.490–2.974, *p* < 0.0001) and real-time PCR (OR: 9.093, 95% CI: 2.163–38.225, *p* < 0.0001). In regard to tissue preservation methods, EBV genome detection was significantly more frequently detected in women with breast cancer for both FFPE specimens (OR: 2.956, 95% CI: 1.608–5.431, *p* < 0.0001) and fresh frozen specimens (OR: 3.371, 95% CI: 1.532–7.419, *p* = 0.003). Moreover, frequency of EBV detection remains significantly higher in breast cancer cases compared to the control group, irrespective of its consistency, namely women with healthy normal breasts (OR: 2.608, 95% CI: 1.218–5.584, *p* = 0.014), women with benign breast diseases (OR: 2.639, 95% CI: 1.103–6.315, *p* = 0.029) and mixed population (OR: 19.019, 95% CI: 3.671–98.532, *p* < 0.0001). Finally, EBV molecular detection was statistically significantly more frequent in breast cancer patients compared with controls across all continents where studies were conducted—Africa (OR: 3.626, 95% CI: 1.623–8.105, *p* = 0.002), Asia (OR: 2.834, 95% CI: 1.508–5.325, *p* = 0.001) and Europe (OR: 15.838, 95% CI: 2.117–118.491, *p* = 0.007)]—except for Oceania (OR: 0.551, 95% CI: 0.190–1.601, *p* = 0.273).

**Figure 9:**
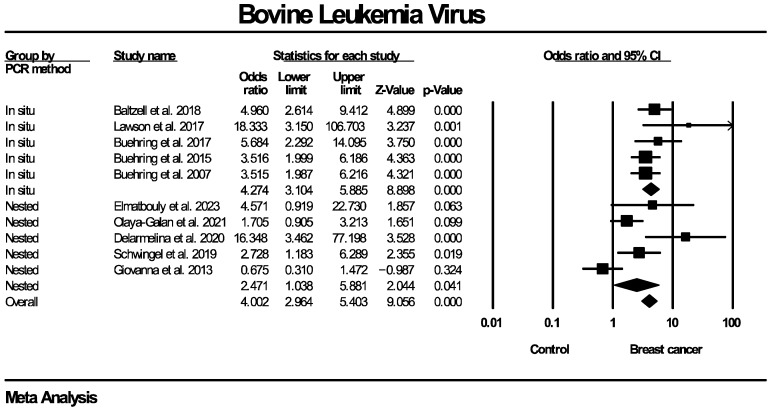
BLV molecular detection—subgroup analysis based on PCR method. Frequency of BLV genome molecular detection was statistically significantly higher in breast cancer patients compared to women without breast malignancies, across all PCR techniques employed (nested and in situ PCR).

**Figure 10:**
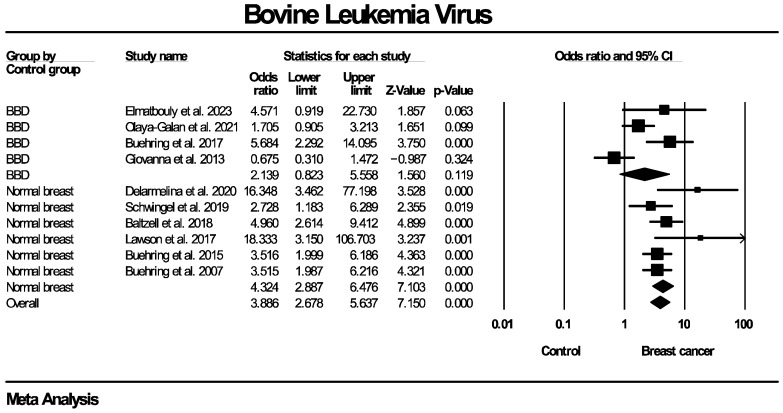
BLV molecular detection—subgroup analysis based on types of control groups. Frequency of BLV genome molecular detection was statistically significantly higher in breast cancer patients compared to women without breast malignancies, across all types of control groups used in individual studies (healthy women with normal breasts, women diagnosed with benign breast diseases). BBD: benign breast disease.

**Figure 11:**
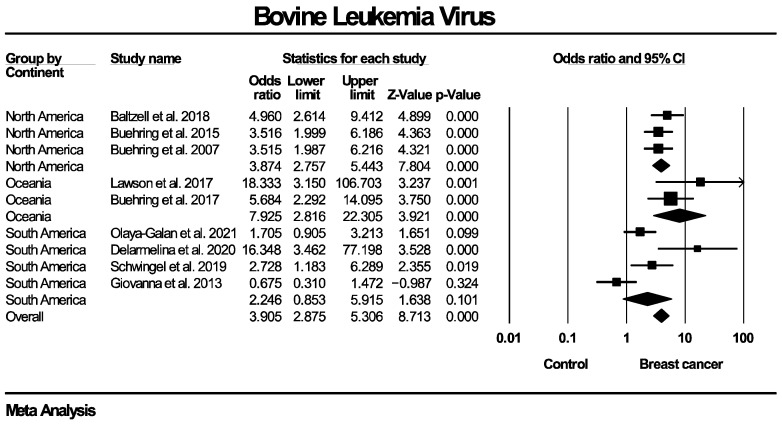
BLV molecular detection—subgroup analysis based on geographical regions/continents of populations enrolled in included studies. Frequency of BLV genome molecular detection was statistically significantly higher in breast cancer patients compared to women without breast malignancies, across all evaluated geographic regions/continents, with the exception of South America.

The forest plots for the subgroup analyses regarding BLV genome detection are presented in Figure 9, Figure 10 and Figure 11. Regarding PCR methodology, the frequency of BLV molecular detection was significantly higher in breast cancer patients compared for both nested PCR (OR: 2.471, 95% CI: 1.038–5.881, *p* = 0.041) and in situ PCR (OR: 4.274, 95% CI: 3.104–5.885, *p* < 0.0001). No subgroup analysis stratified by tissue preservation method was performed because all included studies used FFPE breast tissue. As for the differences in populations enrolled in the control groups, the frequency of BLV detection was significantly higher in breast cancer cases compared to women with normal breasts (OR: 4.324, 95% CI: 2.887–6.476, *p* < 0.0001), but no statistical significance was identified when compared to women with benign breast diseases (OR: 2.139, 95% CI: 0.823–5.558, *p* = 0.029). Additionally, a significantly higher frequency of BLV detection in breast cancer patients compared with controls was observed across studies originating from North America (OR: 3.874, 95% CI: 2.757–5.443, *p* < 0.0001) and Oceania (OR: 7.925, 95% CI: 2.816–22.305, *p* < 0.0001), while no significant association was found for studies from South America (OR: 2.246, 95% CI: 0.853–5.915, *p* = 0.101).

### 3.5. Publication Bias

**Figure 12:**
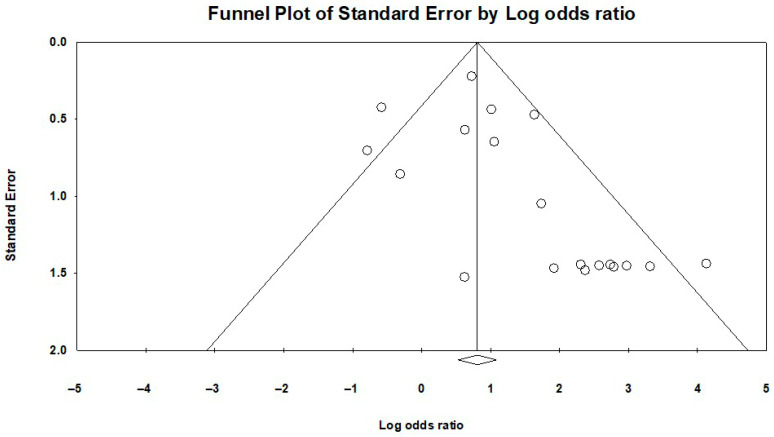
EBV molecular detection funnel plot—publication bias. The funnel plot is asymmetrical, which indicates the presence of publication bias.

Both outcome measures of the present meta-analysis contained data from at least 10 studies; therefore, publication bias was assessed separately for each outcome. The funnel plot corresponding to molecular detection of the EBV genome is presented in Figure 12. Visual inspection of the plot revealed asymmetry, indicating the need for further investigation of potential publication bias with appropriate statistical tests. The results of Egger’s regression test (1-tailed *p*-value = 0.01433; 2-tailed *p*-value = 0.02867) suggested the presence of publication bias, so Duval and Tweedie’s trim-and-fill test was used to assess whether potential omission of studies due to publication bias could have altered the pooled effect estimate for this outcome. Assuming that a number of studies were missing to the right of the axis representing the pooled odds ratio in the funnel plot, the adjusted OR and 95% CI were identical to those previously reported, indicating that no studies are missing on this direction. However, when assuming that studies were missing to the left of the axis, the adjusted effect estimate was OR: 1.96390 with a 95% CI of 1.15185–3.34844, suggesting that seven studies may be missing on the left side of the plot. Although the adjusted OR differed numerically from the original estimate, it retained the same direction of effect and remained statistically significant, confirming that the frequency of molecular detection of the EBV genome is significantly higher in patients with breast cancer compared with women without breast malignancies.

**Figure 13:**
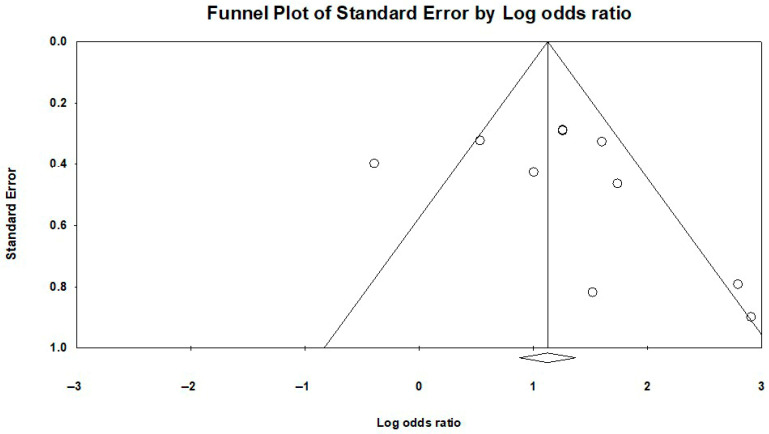
BLV molecular detection funnel plot—publication bias. The funnel plot is asymmetrical, which indicates the presence of publication bias.

The funnel plot corresponding to the outcome of BLV genome molecular detection is presented in Figure 13. Similarly, visual inspection of this funnel plot revealed slight asymmetry, but Egger’s test (1-tailed *p*-value = 0.13524; 2-tailed *p*-value = 0.27047) was not indicative of publication bias. Further investigation using Duval and Tweedie’s trim-and-fill method was conducted to confirm the absence of publication bias. No studies were found missing on the right of the axis representing the pooled odds ratio, but three studies were estimated to be missing from the left side of the funnel plot, as the adjusted effect estimate was OR: 2.66104 with a 95% CI of 2.10909–3.35744. Although different, the result maintained the same direction of effect and remained statistically significant, confirming that the omission of these studies did not lead to a different conclusion regarding the comparison of BLV genome molecular detection between breast cancer patients and control populations.

## 4. Discussion

### 4.1. Main Findings

The synthesis of evidence from observational studies shows that the frequency of molecular detection of EBV and BLV viral genomes was significantly higher in patients with histologically proven breast cancer, compared to healthy controls or women with histologically diagnosed benign breast diseases.

### 4.2. Interpretation of the Results

According to the results of the present meta-analysis, the frequency of molecular detection of the EBV genome was statistically significantly higher in women diagnosed with breast cancer compared with women with healthy breasts or benign breast disease. This finding is of particular importance in the context of evaluating the hypothesized involvement of EBV, through infection of mammary gland cells, in breast carcinogenesis. EBV infection is highly prevalent in humans, with more than 95% of the adult population worldwide estimated to be seropositive for antibodies against the virus [12]. Although no clear geographical distribution pattern has been identified over recent decades, the incidence of breast cancer has been increasing rapidly in Europe and North America, a trend that has been associated with lifestyle changes in Western populations and exposure to environmental factors such as diet, stress, physical activity, occupational habits, alcohol consumption, smoking, and overall lifestyle patterns [12]. However, the high seroprevalence of EBV in the general population renders the investigation of an association between EBV seropositivity and breast cancer development particularly challenging, whereas the presence of the virus within mammary tissue appears to be of greater relevance when exploring its potential role in breast carcinogenesis [50]. The presence of lymphocytic cell populations within the mammary gland makes this tissue susceptible to EBV infection [51]. Multiple mechanisms have been investigated as potential pathways through which EBV infection may induce oncogenesis in breast tissue. A prominent role appears to be played by viral proteins expressed after viral entry into host cells, including Epstein–Barr virus nuclear antigens (EBNAs) and latent membrane proteins (LMPs). These viral proteins dysregulate critical signaling pathways, promoting cellular proliferation while simultaneously compromising antiviral immune responses [12]. Specifically, EBNA-3 proteins (3A, 3B and 3C) contribute to the initiation and survival of malignant cells through inactivation of tumor suppressor genes, whereas the membrane protein LMP-1 mimics the activity of CD40, a member of the tumor necrosis factor (TNF) receptor family, thereby activating multiple signaling pathways implicated in carcinogenesis [52,53]. In addition, EBV has been hypothesized to exert oncogenic effects through activation of the HER2 and HER3 signaling pathways, predisposing mammary epithelial cells to malignant transformation [54]. Finally, delayed primary EBV infection occurring later in life may further contribute to its oncogenic potential, as evidence suggests that it induces a stronger host immune response, even during asymptomatic infection, leading to prolonged immune activation, increased levels of TNF-α and interleukin-6 (IL-6), stimulation of aromatase activity, enhanced conversion of androstenedione to estrogens in adipose tissue, and ultimately an increased risk of breast cancer development [54]. The findings of the present systematic review and meta-analysis, demonstrating significantly increased detection of the EBV genome in breast tissue of women with breast cancer through synthesis of data from multiple individual studies, are consistent with the biological mechanisms previously proposed in the literature, and may strengthen evidence towards the hypothesis that EBV may be biologically associated with breast carcinogenesis. However, these findings should be interpreted cautiously, as tissue-based viral genome detection alone cannot establish a causal relationship between EBV infection and breast cancer development.

Similarly, the frequency of molecular detection of the BLV genome was found to be significantly higher in patients with histologically confirmed breast cancer compared with women without breast malignancies. This finding is particularly intriguing in the context of evaluating the potential role of BLV infection, and specifically the presence of viral genomic material within mammary cells, in the pathogenesis of breast cancer. Transmission of BLV to humans is believed to occur through consumption of dairy products or meat derived from infected cattle [55]. Given the variability in bovine infection prevalence and dietary habits across countries and continents, BLV infection demonstrates a distinctive geographical distribution. BLV receptors are found in a variety of tissues, and viral particles are capable of entering cells of different species following viremia, primarily infecting lymphocytes; however, their ability to enter mammary epithelial cells has also been well documented [56]. BLV establishes lifelong infection in host cells by integrating its genome into host cellular DNA. Consequently, a single viral entry event can result in chronic infection, allowing viral genomic interference with cellular processes even years after the initial infection [57]. Published studies have detected BLV genetic material in cells of precancerous breast lesions that later progressed to invasive malignancies, as well as in histologically normal breast tissue from women who developed breast cancer many years later. These findings strongly suggest viral involvement even at early stages of breast carcinogenesis [46]. Based on these observations, several research groups have classified the presence of BLV in breast tissue among strong risk factors for breast cancer development, emphasizing that its detection may be of greater significance than other established risk factors, such as long-term hormone therapy or a positive family history of breast cancer [18]. The precise mechanisms through which BLV initiates or promotes breast carcinogenesis remain unclear; however, the viral tax gene has been identified as a key mediator of its oncogenic activity. Expression of the tax gene inhibits cellular DNA repair mechanisms, leading to oxidative stress, accumulation of genetic mutations, and promotion of malignant transformation [57]. Furthermore, the presence of BLV genomic material in mammary epithelial cells has been associated with overexpression of cellular proliferation markers Ki-67 and HER2 in patients with breast cancer, suggesting potential involvement of the virus in additional oncogenic pathways [18]. Collectively, the results of the present meta-analysis strengthen existing evidence in the international literature supporting potential contribution of BLV infection in breast carcinogenesis, demonstrating that despite occasionally conflicting findings from individual studies, pooled data consistently reveal a significantly higher frequency of viral detection in women with histologically confirmed breast cancer.

### 4.3. Comparison with Existing Literature

The results of the present study are consistent with those of previous systematic reviews and meta-analyses comparing the frequency of molecular detection of EBV and/or BLV between breast cancer patients and control groups. The systematic review and meta-analysis by Saeedi-Moghaddam et al. [58], published in 2024, demonstrated significantly increased BLV detection in tissue specimens of women with breast cancer. Performed according to PRISMA guidelines and providing a robust publication bias analysis and an interesting interpretation of results based on geographical criteria, this study, however, had some methodological shortcomings, as it neither predefined in its methodology the types of specimens nor accepted methods of genome molecular detection used in included studies; meanwhile, it also did not provide clear eligibility criteria for the control subgroups of the studies. Regarding reported results, the study included studies using mixed types of specimens, namely breast tissue and blood samples; the analyses were conducted using a fixed effects model and no comparisons to previous similar meta-analyses were attempted.

The study by Agolli et al. [54], published in 2023, concluded that the frequency of EBV genome molecular detection was significantly higher in patients with breast malignancies compared to a control group of women without breast malignancies. Although conducted according to PRISMA guidelines, the study did not include a statistical analysis of the individual study results but instead calculated cumulative frequencies of viral detection in the patient and control groups; it did not attempt publication bias assessment, and included studies that performed molecular analysis using various methods apart from PCR (such as immunohistochemical methods and in situ hybridization) on multiple biological materials such as blood serum, blood leukocytes, and human breast milk.

Jin et al. [59] published a systematic review and meta-analysis in 2020 that also reported a significantly higher frequency of EBV molecular detection in women with breast cancer. This study was conducted based on MOOSE guidelines (Meta-analysis of Observational Studies in Epidemiology) and not PRISMA, and its methodology did not predefine whether the statistical analysis would be performed using a random or fixed effects model, noting that this would be decided after heterogeneity assessment. Furthermore, although it exclusively used breast tissue as biological material, the study included individual studies that used molecular analysis methods other than PCR.

The study by Khatami et al. [60], published in 2020, attempted to compare the frequency of BLV molecular detection between breast cancer patients and women without known breast malignancies, finding a significantly higher frequency in the patient group. The study complied with PRISMA guidelines but did not define clear inclusion criteria for the control group, while accepting individual studies in which blood samples, rather than breast tissue, were collected for molecular analysis, which was also performed using multiple different methods.

The systematic review and meta-analysis by Farahmand et al. [12], published in 2019, which focused on comparing EBV molecular detection between groups of women with and without breast cancer, concluded that the detection frequency was significantly higher in patients with breast malignancies. The study, conducted according to PRISMA guidelines, did not only include studies performing molecular analysis in breast tissue but also incorporated studies using blood samples, and did not define a preferred molecular analysis method to detect viral genomes, including studies that performed either PCR, immunohistochemistry, or in situ hybridization.

Huo et al. [61] published a meta-analysis in 2012 that reported a significantly higher frequency of EBV genome detection in women with breast cancer compared to the control group, without clearly defining the composition of the latter group. Although it accepted individual studies that performed molecular analysis exclusively using PCR, the methodology did not predefine the choice of a random or fixed effects model for statistical analysis, noting that this would be decided following heterogeneity assessment, and it did not follow PRISMA guidelines or assess the methodological quality of the included individual studies by using an appropriate scale or evaluation tool.

Finally, the study by Gao et al. [57], published in 2020, investigating the role of BLV in breast carcinogenesis, was effectively a systematic review rather than a meta-analysis, as it presented and evaluated international literature data in detail without including a methodology or statistical analysis corresponding to a meta-analysis.

Regardless of potential methodological limitations or deviations, compared to the present meta-analysis, the fact that the findings of previously published systematic reviews and meta-analyses are in agreement with those of the present study further strengthens the validity of the results presented above.

### 4.4. Strengths and Limitations

The present systematic review and meta-analysis is the first to compare the frequency of molecular detection of EBV and BLV genomes between breast cancer patients and women without known breast malignancies. All previously published related systematic reviews and meta-analyses examined only one of these two viral agents. A key strength of this study is the inclusion of individual studies that evaluated viral genome detection exclusively in breast tissue samples and by PCR methods. This approach ensures greater methodological consistency among the included studies and facilitates interpretation of the results in the context of the potential role of these viruses in breast carcinogenesis, since the presence of the viruses within breast cells is considered a fundamental requirement for their involvement in pathogenic mechanisms of breast cancer, whereas detection in other tissues, such as blood, is insufficient. Moreover, the inclusion of studies that used only PCR-based molecular detection further reduces methodological variability and maximizes viral genome detection rates, thereby minimizing false-negative results that could affect the overall meta-analysis findings.

A notable limitation of this meta-analysis is that the majority of included studies were rated to be of moderate methodological quality, with only a few considered as high quality. While this is expected for observational studies, it may limit the confidence in the findings, and highlights the need for well-designed studies to accurately assess the frequency of EBV and BLV molecular detection in breast cancer patients. However, sensitivity analysis performed for EBV genome detection, restricted to high-quality studies, showed results consistent with the primary analysis, supporting the robustness of the findings. On the contrary, as a similar formal analysis was not feasible for the outcome of BLV molecular detection, the respective results should be more cautiously interpreted. Additionally, this study focused on detecting viral genomes in breast tissue without correlating the results with immunological markers that could indicate whether the infection is past or active, a correlation of clinical relevance that warrants further investigation in future research. Another limitation is the reported heterogeneity in both EBV (I^2^ = 55.5%) and BLV (I^2^ = 70.9%) outcomes, which prompted subgroup analyses to explore potential sources of variability. These analyses demonstrated that the association between viral detection and breast cancer was fairly consistent across different PCR methods, tissue preservation techniques, control group types, and geographic regions, particularly for EBV, suggesting a robust result despite methodological diversity. At the same time, there were clear differences in the size of the effect across subgroups, and some subgroups did not reach statistical significance (for example, Oceania for EBV, benign breast disease controls, and South America for BLV), indicating that these factors may contribute, at least partly, to the observed heterogeneity rather than fully account for it. Notably, the predominance of moderate-quality observational studies likely introduces some residual confounding and methodological variability. Overall, while the direction of association appears stable across analyses, heterogeneity is likely multifactorial and reflects a combination of methodological and clinical differences across studies. Finally, regarding publication bias, funnel plot inspection and Egger’s regression test indicated asymmetry for EBV genome detection. Trim-and-fill analysis resulted in a lower adjusted pooled effect size compared with the original estimate, although the association remained statistically significant and in the same direction. This may indicate that the magnitude of the EBV–breast cancer association may be partially overestimated in the primary analysis, which should be pointed out as it does influence the interpretation of the results. This difference is potentially observed due to publication bias, attributed to the possible omission of grey literature or studies with small sample sizes and either low viral genome detection rates or non-significant results. However, funnel plot asymmetry may also reflect genuine clinical heterogeneity among studies, arising from differences in study design, populations and laboratory methodologies, rather than publication bias alone.

### 4.5. Generalizability and Applicability

Breast cancer is a complex, multifactorial disease with a continuously increasing incidence among women worldwide. Investigating individual etiological factors, particularly viral agents that have been consistently implicated in the development of various types of cancer, may provide a deeper understanding of the pathogenic mechanisms leading to breast carcinogenesis. The present study does not aim to establish, nor can it support, a causal relationship between breast cancer and infection with EBV and BLV. However, it aims to enhance existing knowledge by drawing conclusions regarding the controversial role of EBV and BLV viral infections in breast carcinogenesis and to provide evidence that may be useful to future primary research on this topic, which may, in the long term, contribute to improving clinical practice, prevention strategies, early detection at preclinical stages of carcinogenesis, and developing more effective and personalized therapeutic approaches for breast cancer cases.

However, considering the complex and not fully elucidated pathophysiological mechanisms of the disease, it is rather optimistic to assume that a test of a single biomarker, such as the detection of a viral agent, could accurately diagnose and predict breast cancer development. Such a biomarker is more likely to serve as an adjunct diagnostic tool and a component of personalized treatment planning, in combination with multiple other tools. Specifically, for BLV, which is transmitted through the consumption of products derived from infected cattle, definitive evidence of its involvement in breast carcinogenesis could guide the implementation of systematic testing of cattle for infection and of animal-derived products for viral contamination as a public health measure to minimize human exposure and, consequently, breast cancer risk. It could also support the establishment of regular or more systematic pre-symptomatic screening programs for subpopulations in contact with cattle who constitute high-risk groups.

Gaining insights from high-scale research efforts investigating other biomarkers will significantly contribute to clarifying the role and potential exploitation of molecular detection of EBV and BLV in the prevention, diagnosis, and management of breast cancer. It should be noted, however, that the limitations of the present study, as previously outlined, make the generalization of its findings to routine clinical practice relatively premature. Before introducing any biomarker into everyday clinical practice, a rigorous evaluation of the validity of its results, its utility in patient management decisions, and the associated costs, is essential. Therefore, further research is required to support the use of molecular detection of EBV and BLV genomes in breast cancer management, in the form of methodologically robust studies that will allow future systematic reviews and diagnostic accuracy meta-analyses to generate more reliable, generalizable, and clinically applicable conclusions.

## 5. Conclusions

The present systematic review and meta-analysis concluded that the frequency of molecular detection of the EBV and BLV viral genomes is significantly higher in patients with histologically confirmed breast cancer compared to women with healthy breasts or patients with histologically confirmed benign breast diseases. The presence of these viral agents in breast tissue could potentially play a role in breast carcinogenesis, while molecular detection of their genomes could potentially be utilized in the future for preventive, diagnostic, and therapeutic purposes. Further research, namely carefully designed and methodologically robust studies, is required to fully clarify any direct or indirect involvement of EBV and BLV in breast oncogenesis and their potential role in the management of breast cancer patients, as no evidence-based causal relationship between viral infection and breast cancer development has yet been established by existing literature.

## Figures and Tables

**Table 1:** Characteristics of included studies.

Study	Country	Study Type	Patients with Breast Cancer *(n)*	Control Group *(n)*	Intervention	Virus	Characteristics of Breast Cancer Patients (Case Group)	Characteristics of Women Without Breast Malignancy (Control Group)	Outcomes
Khasawneh et al. 2024 [25]	Jordan	Retrospective case–control study	110	30	Molecular detection of viral genome with real time PCR in formalin-fixed paraffin-embedded breast tissue	EBV	Inclusion criteria: patients with histologically diagnosed breast cancer, aged 20–80 years, positivity in GAPDH in PCR Εxclusion criteria: neoadjuvant chemotherapy or radiotherapy, no available information on molecular subtype	Inclusion criteria: patients with histologically diagnosed benign breast diseases Exclusion criteria: -	Comparison of EBV genome molecular detection between patients with breast cancer and women with benign breast diseases
Elmatbouly et al. 2023 [42]	Egypt	Retrospective case–control study	50	50	Molecular detection of viral genome with nested PCR in formalin-fixed paraffin-embedded breast tissue	BLV	Inclusion criteria: patients with histologically diagnosed breast cancer Exclusion criteria: -	Inclusion criteria: patients with histologically diagnosed benign breast diseases Exclusion criteria: -	Comparison of BLV genome molecular detection between patients with breast cancer and women with benign breast diseases
Gihbid et al. 2023 [26]	Morocco	Retrospective case–control study	76	12	Molecular detection of viral genome with PCR in breast biopsy tissue stored in liquid nitrogen (fresh frozen tissue)	EBV	Inclusion criteria: patients with histologically diagnosed breast cancer Exclusion criteria: -	Inclusion criteria: patients with histologically diagnosed breast fibroadenoma Exclusion criteria: -	Comparison of EBV genome molecular detection between patients with breast cancer and women with benign breast diseases
Gupta et al. 2022 [27]	Qatar	Case–control study	74	14	Molecular detection of viral genome with PCR in formalin-fixed paraffin-embedded breast tissue	EBV	Inclusion criteria: patients with histologically diagnosed breast cancer Exclusion criteria: neoadjuvant chemotherapy, radiotherapy, hormonal therapy, immunotherapy or other targeted therapy	Inclusion criteria: women with healthy breasts or women with histologically diagnosed benign breast diseases Exclusion criteria: -	Comparison of EBV genome molecular detection between patients with breast cancer and women with healthy breasts or benign breast diseases
Gupta et al. 2021 [28]	Croatia	Case–control study	70	14	Molecular detection of viral genome with PCR in formalin-fixed paraffin-embedded breast tissue	EBV	Inclusion criteria: patients with histologically diagnosed triple negative breast cancer Exclusion criteria: -	Inclusion criteria: women with healthy breasts or women with histologically diagnosed benign breast diseases Exclusion criteria: -	Comparison of EBV genome molecular detection between patients with breast cancer and women with healthy breasts or benign breast diseases
Nagi et al. 2021 [29]	Lebanon	Case–control study	102	14	Molecular detection of viral genome with PCR (followed by detection with immunohistochemical methods) in formalin-fixed paraffin-embedded breast tissue	EBV	Inclusion criteria: patients with histologically diagnosed breast cancer Exclusion criteria: neoadjuvant chemotherapy, radiotherapy, hormonal therapy, immunotherapy, other targeted therapy, men, women of other ethnicities apart from Lebanese	Inclusion criteria: women with healthy breasts Exclusion criteria: -	Comparison of EBV genome molecular detection between patients with breast cancer and women with healthy breasts
Olaya-Galán et al. 2021 [43]	Colombia	Retrospective case–control study	75	83	Molecular detection of viral genome mainly with nested- liquid PCR and secondarily with in situ PCR (followed by detection with immunohistochemical methods) in formalin-fixed paraffin-embedded breast tissue	BLV	Inclusion criteria: patients with histologically diagnosed breast cancer, aged > 18 years, with lesions > 4 mm in diameter and sufficient tissue specimens for pathological examination and molecular analysis Exclusion criteria: tissue specimen composed mainly of adipose tissue, specimen insufficient/ineligible for molecular analysis	Inclusion criteria: women with histologically diagnosed benign breast diseases, aged > 18 years, with lesions > 4 mm in diameter and sufficient tissue specimens for pathological examination and molecular analysis Exclusion criteria: tissue specimen composed mainly of adipose tissue, specimen insufficient/ineligible for molecular analysis	Comparison of BLV genome molecular detection between patients with breast cancer and women with benign breast diseases
Al Hammad et al. 2020 [30]	Jordan	Case–control study	100	20	Molecular detection of viral genome with real-time PCR in formalin-fixed paraffin-embedded breast tissue	EBV	Inclusion criteria: patients with histologically diagnosed breast cancer Exclusion criteria: -	Inclusion criteria: women with healthy breasts that underwent breast reduction surgery Exclusion criteria: -	Comparison of EBV genome molecular detection between patients with breast cancer and women with healthy breasts
Delarmelina et al. 2020 [44]	Brazil	Retrospective case–control study	49	39	Molecular detection of viral genome with nested and semi-nested PCR in formalin-fixedparaffin-embedded breast tissue	BLV	Inclusion criteria: patients with histologically diagnosed breast cancer Exclusion criteria: neoadjuvant chemotherapy, radiotherapy, hormonal therapy	Inclusion criteria: women with healthy breasts that underwent breast reduction surgery Exclusion criteria: -	Comparison of BLV genome molecular detection between patients with breast cancer and women with healthy breasts
Mostafaei et al. 2020 [31]	Iran	Multicenter case–control study	83	31	Molecular detection of viral genome with nested PCR in fresh frozen breast tissue	EBV	Inclusion criteria: patients with histologically diagnosed breast cancer Exclusion criteria: present or previous personal history of chemotherapy and/or radiotherapy, anticancer therapy with biological agents, current pregnancy, systematic inflammatory disease	Inclusion criteria: women with healthy breasts, age-matched with breast cancer patients Exclusion criteria: personal history of estrogen-based hormonal therapy, use of contraceptive pills, cervical cancer, smoking	Comparison of EBV genome molecular detection between patients with breast cancer and women with healthy breasts
Mofrad et al. 2020 [32]	Iran	Case–control study	59	11	Molecular detection of viral genome with PCR in formalin-fixed paraffin-embedded breast tissue	EBV	Inclusion criteria: patients with histologically diagnosed breast cancer Exclusion criteria: -	Inclusion criteria: women with healthy breasts Exclusion criteria: -	Comparison of EBV genome molecular detection between patients with breast cancer and women with healthy breasts
Sharifpour et al. 2019 [33]	Iran	Retrospective case–control study	37	35	Molecular detection of viral genome with nested PCR in formalin-fixed paraffin-embedded breast tissue	EBV	Inclusion criteria: patients with histologically diagnosed breast cancer Exclusion criteria: -	Inclusion criteria: women histologically diagnosed breast fibroadenoma Exclusion criteria: -	Comparison of EBV genome molecular detection between patients with breast cancer and women with benign breast diseases
Karbalaie Niya et al. 2019 [34]	Iran	Case–control study	210	198	Molecular detection of viral genome with typical and nestedPCR in paraffin-embedded breast tissue	EBV	Inclusion criteria: patients with histologically diagnosed breast cancer Exclusion criteria: -	Inclusion criteria: women with healthy breasts Exclusion criteria: -	Comparison of EBV genome molecular detection between patients with breast cancer and women with healthy breasts
Dowran et al. 2019 [24]	Iran	Case–control study	150	150	Molecular detection of viral genome within-house PCR in paraffin-embedded breast tissue	EBV	Inclusion criteria: patients with histologically diagnosed breast cancer Exclusion criteria: -	Inclusion criteria: women with healthy breasts or women with histologically diagnosed breast fibroadenoma, fibrocystic disease or adenosis Exclusion criteria: -	Comparison of EBV genome molecular detection between patients with breast cancer and women with healthy breasts or benign breast diseases
Schwingel et al. 2019 [45]	Brazil	Retrospective case–control study	72	72	Molecular detection of viral genome with nested PCR in formalin-fixed paraffin-embedded breast tissue	BLV	Inclusion criteria: patients with histologically diagnosed breast cancer Exclusion criteria: -	Inclusion criteria: women with healthy breasts that underwent breast reduction surgery Exclusion criteria: -	Comparison of BLV genome molecular detection between patients with breast cancer and women with healthy breasts
Baltzell et al. 2018 [46]	United States of America	Retrospective case–control study	90	103	Molecular detection of viral genome with in situ PCR in formalin-fixed paraffin-embedded breast tissue	BLV	Inclusion criteria: patients with histologically diagnosed breast cancer, aged > 18 years Exclusion criteria: insufficient/damaged tissue specimen, lack of epithelial cells in tissue specimen	Inclusion criteria: women with healthy breasts, aged > 18 years Exclusion criteria: insufficient/damaged tissue specimen, lack of epithelial cells in tissue specimen	Comparison of BLV genome molecular detection between patients with breast cancer and women with healthy breasts
Fessahaye et al. 2017 [35]	Eritrea	Retrospective case–control study	114	63	Molecular detection of viral genome with PCR (followed by detection with immunohistochemical methods and in situ hybridization in formalin-fixed paraffin-embedded breast tissue	EBV	Inclusion criteria: patients with histologically diagnosed breast cancer Exclusion criteria: -	Inclusion criteria: women with histologically diagnosed breast fibroadenoma Exclusion criteria: -	Comparison of EBV genome molecular detection between patients with breast cancer and women with benign breast diseases
El-Naby et al. 2017 [36]	Egypt	Retrospective case–control study	42	42	Molecular detection of viral genome with nested PCR (followed by detection with immunohistochemical methods) in paraffin-embedded breast tissue	EBV	Inclusion criteria: patients with histologically diagnosed breast cancer Exclusion criteria: -	Inclusion criteria: women with histologically diagnosed breast fibroadenoma Exclusion criteria: -	Comparison of EBV genome molecular detection between patients with breast cancer and women with benign breast diseases
Lawson et al. 2017 [8]	Australia	Retrospective case–control study	12 (EBV) 22 (BLV)	16 (EBV) 17 (BLV)	Molecular detection of viral EBV genome with typical and nested PCR in formalin-fixed specimens of breast tissue and molecular detection of viral BLV genome with typical and in situ PCR in formalin-fixed specimens of breast tissue	EBV & BLV	Inclusion criteria: patients with histologically diagnosed breast cancer and previous breast biopsy without pathological findings indicative of malignancy Exclusion criteria: -	Inclusion criteria: women with healthy breasts that underwent breast reduction surgery Exclusion criteria: -	Comparison of EBV genome molecular detection between patients with breast cancer and women with healthy breasts Comparison of BLV genome molecular detection between patients with breast cancer and women with healthy breasts
Buehring et al. 2017 [47]	Australia	Retrospective case–control study	50	46	Molecular detection of viral genome with in situ PCR in formalin-fixed paraffin-embedded breast tissue	BLV	Inclusion criteria: patients with histologically diagnosed breast cancer Exclusion criteria: insufficient/damaged tissue specimen, lack of epithelial cells in tissue specimen	Inclusion criteria: women with histologically diagnosed benign breast diseases Exclusion criteria: -	Comparison of BLV genome molecular detection between patients with breast cancer and women with benign breast diseases
Naushad et al. 2016 [37]	Pakistan	Case–control study	250	15	Molecular detection of viral genome with PCR in formalin-fixed paraffin-embedded breast tissue	EBV	Inclusion criteria: patients with histologically diagnosed invasive breast cancer or in situ ductal breast carcinoma Exclusion criteria: -	Inclusion criteria: women with healthy breasts Exclusion criteria: -	Comparison of EBV genome molecular detection between patients with breast cancer and women with healthy breasts
Buehring et al. 2015 [16]	United States of America	Retrospective case–control study	114	104	Molecular detection of viral genome with in situ PCR in formalin-fixed paraffin-embedded breast tissue	BLV	Inclusion criteria: patients with histologically diagnosed breast cancer Exclusion criteria: -	Inclusion criteria: women with healthy breasts—tissue specimens were mainly obtained by women that underwent breast reduction surgery Exclusion criteria: -	Comparison of BLV genome molecular detection between patients with breast cancer and women with healthy breasts
Giovanna et al. 2013 [17]	Colombia	Case–control study	53	53	Molecular detection of viral genome with nested PCR paraffin-embedded breast tissue	BLV	Inclusion criteria: patients with histologically diagnosed breast cancer Exclusion criteria: -	Inclusion criteria: women with histologically diagnosed benign breast disease Exclusion criteria: -	Comparison of BLV genome molecular detection between patients with breast cancer and women with benign breast diseases
Zekri et al. 2012 [38]	Egypt and Iraq	Case–control study	90	40	Molecular detection of viral genome with PCR (followed by detection with immunohistochemical methods and in situ hybridization) in paraffin-embedded breast tissue	EBV	Inclusion criteria: patients with histologically diagnosed breast cancer Exclusion criteria: personal history of previous breast cancer or other malignancy	Inclusion criteria: women with healthy breasts Exclusion criteria: -	Comparison of EBV genome molecular detection between patients with breast cancer and women with healthy breasts
Glenn et al. 2012 [39]	Australia	Retrospective case–control study	27	18	Molecular detection of viral genome with in situ PCR (followed by detection with immunohistochemical methods) in formalin-fixed breast tissue	EBV	Inclusion criteria: patients with histologically diagnosed invasive breast cancer or in situ ductal breast carcinoma Exclusion criteria: -	Inclusion criteria: women with healthy breasts that underwent breast reduction surgery Exclusion criteria: -	Comparison of EBV genome molecular detection between patients with breast cancer and women with healthy breasts
Mazouni et al. 2011 [13]	France	Retrospective case–control study	196	15	Molecular detection of viral genome with quantitative/real-time PCR in frozen tissue from breast biopsy	EBV	Inclusion criteria: patients with histologically diagnosed breast cancer Exclusion criteria: -	Inclusion criteria: women with healthy breasts that underwent breast reduction surgery or women with histologically diagnosed breast fibroadenoma, phyllodes tumors or breast fibrocystic disease Exclusion criteria: -	Comparison of EBV genome molecular detection between patients with breast cancer and women with healthy breasts or benign breast diseases
Fawzy et al. 2008 [40]	Egypt	Case–control study	40	20	Molecular detection of viral genome with PCR (followed by detection with immunohistochemical methods) in paraffin-embedded breast tissue	EBV	Inclusion criteria: patients with histologically diagnosed unilateral breast cancer that underwent modified radical mastectomy Exclusion criteria: other types of primary cancer, neoadjuvant chemotherapy	Inclusion criteria: women with histologically diagnosed breast fibrocystic disease, age-matched with patients with breast cancer Exclusion criteria: -	Comparison of EBV genome molecular detection between patients with breast cancer and women with benign breast diseases
Buehring et al. 2007 [48]	United States of America	Retrospective case–control study	110	113	Molecular detection of viral genome with in situ PCR in formalin-fixed breast tissue	BLV	Inclusion criteria: patients with histologically diagnosed breast cancer Exclusion criteria: -	Inclusion criteria: women with healthy breasts Exclusion criteria: personal history of breast cancer	Comparison of BLV genome molecular detection between patients with breast cancer and women with healthy breasts
Kalkan et al. 2005 [41]	Turkey	Case–control study	57	55	Molecular detection of viral genome with PCR in formalin-fixed paraffin-embedded breast tissue	EBV	Inclusion criteria: patients with histologically diagnosed breast cancer Exclusion criteria: -	Inclusion criteria: women with histologically diagnosed breast fibroadenoma, granulomatous mastitis, simple hyperplasia or breast fibrocystic disease Exclusion criteria: -	Comparison of EBV genome molecular detection between patients with breast cancer and women with benign breast diseases

Footnotes: EBV: Epstein Barr Virus, BLV: Bovine Leukemia Virus, GAPDH: Glyceraldehyde-3-Phosphate Dehydrogenase.

**Table 2:** Quality assessment of individual studies.

Newcastle–Ottawa Quality Assessment Scale for Case–Control Studies											
Study ID	Selection				Comparability		Exposure			Total	Quality
	Definition of Cases (max:1✵)	Representa-tiveness of Cases (max:1✵)	Selection of Controls (max:1✵)	Definition of Controls (max:1✵)	On Age (max:1✵)	On any Additional Factor (max:1✵)	Ascertain-ment of Exposure (max:1✵)	Same Method of Ascertain-ment for Cases and Controls (max:1✵)	Non-Response Rate (max:1✵)	Total Numbers of Stars (max:9✵)	High: 7–9✵ Moderate: 4–6✵ Low: 0–3✵
Khasawneh 2024 [25]	**✵**	**✵**	-	**✵**	-	-	**✵**	**✵**	**✵**	6	Moderate
Elmatbouly 2023 [42]	**✵**	**✵**	-	**✵**	-	-	**✵**	**✵**	**✵**	6	Moderate
Gihbid 2023 [26]	**✵**	**✵**	-	**✵**	-	-	**✵**	**✵**	**✵**	6	Moderate
Gupta 2022 [27]	**✵**	**✵**	-	**✵**	-	-	**✵**	**✵**	**✵**	6	Moderate
Gupta 2021 [28]	**✵**	**✵**	-	**✵**	-	-	**✵**	**✵**	**✵**	6	Moderate
Nagi 2021 [29]	**✵**	**✵**	-	-	-	-	**✵**	**✵**	**✵**	5	Moderate
Olaya-Galán 2021 [43]	**✵**	**✵**	-	**✵**	-	-	**✵**	**✵**	**✵**	6	Moderate
Al Hammad 2020 [30]	**✵**	**✵**	-	**✵**	-	-	**✵**	**✵**	**✵**	6	Moderate
Delarmelina 2020 [44]	**✵**	**✵**	-	**✵**	-	-	**✵**	**✵**	**✵**	6	Moderate
Mostafaei 2020 [31]	**✵**	**✵**	**✵**	**✵**	**✵**	-	**✵**	**✵**	**✵**	8	High
Mofrad 2020 [32]	**✵**	**✵**	**✵**	**✵**	-	-	**✵**	**✵**	**✵**	7	High
Sharifpour 2019 [33]	**✵**	**✵**	-	**✵**	-	-	**✵**	**✵**	**✵**	6	Moderate
Karbalaie Niya 2019 [34]	**✵**	**✵**	**✵**	**✵**	-	-	**✵**	**✵**	**✵**	7	High
Dowran 2019 [24]	**✵**	**✵**	-	**✵**	-	-	**✵**	**✵**	**✵**	6	Moderate
Schwingel 2019 [45]	**✵**	**✵**	-	**✵**	-	-	**✵**	**✵**	**✵**	6	Moderate
Baltzell 2018 [46]	**✵**	**✵**	-	**✵**	-	-	**✵**	**✵**	**✵**	6	Moderate
Fessahaye 2017 [35]	**✵**	**✵**	-	**✵**	-	-	**✵**	**✵**	**✵**	6	Moderate
El-Naby 2017 [36]	**✵**	**✵**	-	**✵**	-	-	**✵**	**✵**	**✵**	6	Moderate
Lawson 2017 [8]	**✵**	**✵**	-	**✵**	-	-	**✵**	**✵**	**✵**	6	Moderate
Buehring 2017 [47]	**✵**	**✵**	-	**-**	-	-	**✵**	**✵**	**✵**	5	Moderate
Naushad 2016 [37]	**✵**	**✵**	-	**✵**	-	-	**✵**	**✵**	**✵**	6	Moderate
Buehring 2015 [16]	**✵**	**✵**	-	**✵**	-	-	**✵**	**✵**	**✵**	6	Moderate
Giovanna 2013 [17]	**✵**	**✵**	-	-	-	-	**✵**	**✵**	**✵**	5	Moderate
Zekri 2012 [38]	**✵**	**✵**	**✵**	**✵**	-	-	**✵**	**✵**	**✵**	7	High
Glenn 2012 [39]	**✵**	**✵**	-	**✵**	-	-	**✵**	**✵**	**✵**	6	Moderate
Mazouni 2011 [13]	**✵**	**✵**	-	**✵**	-	-	**✵**	**✵**	**✵**	6	Moderate
Fawzy 2008 [40]	**✵**	**✵**	-	**✵**	**✵**	-	**✵**	**✵**	**✵**	7	High
Buehring 2007 [48]	**✵**	**✵**	**✵**	**✵**	-	-	**✵**	**✵**	**✵**	7	High
Kalkan 2005 [41]	**✵**	**✵**	-	**✵**	-	-	**✵**	**✵**	**✵**	6	Moderate

Footnote: The Newcastle–Ottawa Scale (NOS) is a star-based system used for the assessment of methodological quality. The star symbol ✵ used in the table indicates that the respective criterion has been met for the given study in each column.

**Table 3:** Results of individual studies for outcomes.

Study	EBV Molecular Detection	BLV Molecular Detection
Khasawneh et al. 2024 [25]	5.674 (0.726 to 44.361)	-
Gihbid et al. 2023 [26]	6.818 (0.383 to 121.295)	-
Gupta et al. 2022 [27]	27.494 (1.582 to 477.839)	-
Gupta et al. 2021 [28]	16.253 (0.930 to 283.962)	-
Nagi et al. 2021 [29]	19.569 (1.136 to 337.143)	-
Al Hammad et al. 2020 [30]	13.131 (0.766 to 225.206)	-
Mostafaei et al. 2020 [31]	2.755 (1.169 to 6.491)	-
Mofrad et al. 2020 [32]	1.865 (0.094 to 37.082)	-
Sharifpour et al. 2019 [33]	2.870 (0.807 to 10.212)	-
Karbalaie Niya et al. 2019 [34]	2.068 (1.337 to 3.200)	-
Dowran et al. 2019 [24]	-	-
Fessahaye et al. 2017 [35]	5.135 (2.036 to 12.950)	-
El-Naby et al. 2017 [36]	1.875 (0.613 to 5.738)	-
Lawson et al. 2017 [8]	0.733 (0.137 to 3.938)	-
Naushad et al. 2016 [37]	10.061 (0.593 to 170.621)	-
Zekri et al. 2012 [38]	62.126 (3.705 to 1041.767)	-
Glenn et al. 2012 [39]	0.455 (0.114 to 1.806)	-
Mazouni et al. 2011 [13]	15.441 (0.910 to 262.105)	-
Fawzy et al. 2008 [40]	10.723 (0.587 to 195.907)	-
Kalkan et al. 2005 [41]	0.560 (0.244 to 1.286)	-
Elmatbouly et al. 2023 [42]	-	4.571 (0.919 to 22.730)
Olaya-Galán et al. 2021 [43]	-	1.705 (0.905 to 3.213)
Delarmelina et al. 2020 [44]	-	16.348 (3.462 to 77.198)
Schwingel et al. 2019 [45]	-	2.728 (1.183 to 6.289)
Baltzell et al. 2018 [46]	-	4.960 (2.614 to 9.412)
Lawson et al. 2017 [8]	-	18.333 (3.150 to 106.703)
Buehring et al. 2017 [47]	-	5.684 (2.292 to 14.095)
Buehring et al. 2015 [16]	-	3.516 (1.999 to 6.186)
Giovanna et al. 2013 [17]	-	0.675 (0.310 to 1.472)
Buehring et al. 2007 [48]	-	3.515 (1.987 to 6.216)

The results are presented in the form of odds ratios (ORs) and 95% confidence intervals (95% CIs). Note: In the study by Dowran et al., no EBV genomic material was detected in either the breast cancer group (0/150) or the control group (0/150), so no positive events were available for comparison between groups. Consequently, the odds ratio and corresponding 95% confidence interval could not be calculated due to the absence of events in both groups, since estimation of these measures requires the presence of at least one event in one of the study groups. Therefore, no corresponding OR or 95% CI value is reported in the table.

## Data Availability

No new data were created or analyzed in this study. Data sharing is not applicable to this article.

## Supplementary Materials

The following supporting information can be downloaded at: https://www.mdpi.com/article/10.3390/ijms27104452/s1.

## Abbreviations

The following abbreviations are used in this manuscript:

BBDs	Benign breast diseases
BLV	Bovine Leukemia Virus
CENTRAL	Cochrane Central Register of Controlled Trials
CI 95%	Confidence Interval 95%
DNA	Deoxyribonucleic Acid
EBNA	Epstein–Barr virus nuclear antigens
EBV	Epstein–Barr Virus
ELISA	Enzyme-linked immunosorbent assay
FFPE	Formalin-fixed paraffin-embedded
GAPDH	Glyceraldehyde-3-Phosphate Dehydrogenase
HER	Human epidermal growth factor receptor
IL-6	Interleukin 6
HPV	Human Papilloma Virus
LMP	Latent membrane protein
MMTV	Mouse Mammary Tumor Virus
MOOSE	Meta-analysis of Observational Studies in Epidemiology
NOS	Newcastle–Ottawa Scale
OR	Odds Ratio
PCR	Polymerase Chain Reaction
PRISMA	Preferred Reporting Items for Systematic Reviews and Meta-Analyses
TNF	Tumor Necrosis Factor
