# Supplementary material for: Viral Genomic Footprints in Breast Cancer: A Systematic Review and Meta-Analysis of Tissue-Based Detection of Epstein–Barr Virus and Bovine Leukemia Virus
Source: Int J Mol Sci. 2026 May 15;27(10):4452. doi: 10.3390/ijms27104452 (PMC13206969; doi:10.3390/ijms27104452)
Supplement: Supplementary file 1 [file ijms-27-04452-s001.zip › Supplementary file S2.pdf]

SEARCH STRATEGIES

SEARCH STRING IN MEDLINE DATABASE

(breast cancer[Title/Abstract] OR breast carcinoma[Title/Abstract] OR breast tumor[Title/Abstract] OR breast tumour [Title/Abstract] OR breast neoplasm[Title/Abstract]) AND (Epstein-Barr[Title/Abstract] OR Epstein Barr[Title/Abstract] OR EBV[Title/Abstract])

(breast cancer[Title/Abstract] OR breast carcinoma[Title/Abstract] OR breast tumor[Title/Abstract] OR breast tumour[Title/Abstract] OR breast neoplasm[Title/Abstract]) AND (bovine leukemia virus[Title/Abstract] OR BLV[Title/Abstract])

SEARCH STRING IN SCOPUS DATABASE

(TITLE-ABS-KEY (breast AND cancer OR breast AND carcinoma OR breast AND tumor OR breast AND tumour OR breast AND neoplasm) AND TITLE-ABS-KEY (Epstein-Barr OR Epstein AND Barr OR EBV))

(TITLE-ABS-KEY (breast AND cancer OR breast AND carcinoma OR breast AND tumor OR breast AND tumour OR breast AND neoplasm) AND TITLE-ABS-KEY (Bovine AND Leukemia AND virus OR BLV))

SEARCH STRING IN COCHRANE CENTRAL DATABASE

(breast cancer OR breast carcinoma OR breast tumor OR breast tumour OR breast neoplasm) AND (Epstein Barr virus OR Epstein-Barr OR EBV) in Title Abstract Keyword - (Word variations have been searched)

(breast cancer OR breast carcinoma OR breast tumor OR breast tumour OR breast neoplasm) AND (Bovine Leukemia virus OR BLV) in Title Abstract Keyword - (Word variations have been searched)

SEARCH STRING IN CLINICALTRIALS.GOV DATABASE

Breast Cancer OR Breast Carcinoma OR Breast Tumor OR Breast Tumour OR Breast Neoplasm | All studies AND Epstein-Barr Virus OR Epstein Barr OR EBV | All studies

Breast Cancer OR Breast Carcinoma OR Breast Tumor OR Breast Tumour OR Breast Neoplasm | All studies AND Bovine Leukemia virus OR BLV | All studies
