# Supplementary material for: Viral Genomic Footprints in Breast Cancer: A Systematic Review and Meta-Analysis of Tissue-Based Detection of Epstein–Barr Virus and Bovine Leukemia Virus
Source: Int J Mol Sci. 2026 May 15;27(10):4452. doi: 10.3390/ijms27104452 (PMC13206969; doi:10.3390/ijms27104452)
Supplement: Supplementary file 1 [file ijms-27-04452-s001.zip › Supplementary file S3.pdf]

STUDIES EXCLUDED WITH REASONS

#	Author - year	PMID	Reason of exclusion
1.	Richardson 2015	25723522	EBV molecular detection in different biological materials (serum)
2.	Mashaly 2022	35225477	EBV molecular detection in different biological materials (serum)
3.	Zhang 2022	35842661	EBV molecular detection with other methods apart from PCR
4.	Gouadfel 2023	38158663	Dependent samples- control samples consisted of histologically normal breast tissue collected from the peripheral margins of malignant breast tumors
5.	Hsu 2024	38495506	Molecular detection of EBV genome only in breast cancer patients – lack of control group
6.	Zhang 2017	28928885	EBV molecular detection in different biological materials (serum)
7.	Cox 2010	20407437	EBV molecular detection in different biological materials (serum)
8.	Abdallah 2018	30151354	Other type of molecular analysis (methylation study) - no molecular detection of EBV genome
9.	He 2011	21724319	Serological EBV antibody detection – no molecular detection of EBV genome
10.	Gupta 2021	33006291	Molecular detection of EBV genome only in breast cancer patients – lack of control group
11.	Horiuchi 1994	8063937	Molecular detection of EBV genome only in breast cancer patients – lack of control group
12.	Yasui 2001	11205495	Molecular detection of EBV genome only in breast cancer patients – lack of control group

13.	Mazouni 2015	25798256	Molecular detection of EBV genome only in breast cancer patients – lack of control group
14.	Marrão 2014	25213133	EBV molecular detection in different biological materials (serum) in the control group
15.	Richardson 2004	15150559	EBV molecular detection in different biological materials (serum)
16.	Al Moustafa 2016	27082145	Molecular detection of EBV genome only in breast cancer patients – lack of control group
17.	Salih 2022	35478129	Molecular detection of EBV genome only in breast cancer patients – lack of control group
18.	Ballard 2015	26481274	Molecular detection of EBV genome only in breast cancer patients – lack of control group
19.	Joshi 2009	19997605	EBV molecular detection with other methods apart from PCR
20.	Qi 2014	25010836	Serological EBV antibody detection – no molecular detection of EBV genome
21.	Morales-Sánchez 2013	24131889	Dependent samples- control samples consisted of histologically normal breast tissue collected from the peripheral margins of malignant breast tumors
22.	Wee 2017	28093081	Retrospective-epidemiological stud
23.	Aboulkassim 2015	25933186	Molecular detection of EBV genome only in breast cancer patients – lack of control group
24.	Glaser 2017	28229344	Molecular detection of EBV genome only in breast cancer patients – lack of control group
25.	Heng 2022	35347575	Epidemiological study – no molecular detection of EBV genome
26.	Glenn 2012	22937830	EBV molecular detection in different biological materials (breastmilk)

27.	Yahia 2014	24607238	Dependent samples- control samples consisted of histologically normal breast tissue collected from the peripheral margins of malignant breast tumors
28.	Agborsangaya 2011	20691583	EBV molecular detection in different biological materials (serum)
29.	Mohammadizadeh 2014	25161988	Dependent samples- control samples consisted of histologically normal breast tissue collected from the peripheral margins of malignant breast tumors
30.	Perkins 2006	17163997	Dependent samples- control samples consisted of serum collected from breast cancer patients
31.	Perrigoue 2005	15824148	Dependent samples- control samples consisted of histologically normal breast tissue collected from the peripheral margins of malignant breast tumors
32.	Luqmani 1995	21556618	Molecular detection of EBV genome only in breast cancer patients – lack of control group
33.	Shahi 2022	36444584	Molecular detection of EBV genome only in breast cancer patients – lack of control group
34.	Charostad 2021	34567186	Dependent samples- control samples consisted of histologically normal breast tissue collected from the peripheral margins of malignant breast tumors
35.	Khan 2011	21600202	EBV molecular detection with other methods apart from PCR
36.	Pai 2018	28557251	EBV molecular detection with other methods apart from PCR
37.	Mekrazi 2023	37116366	Molecular detection of EBV genome only in breast cancer patients – lack of control group
38.	Baltzell 2012	22042367	Molecular detection of EBV genome only in breast cancer patients – lack of control group
39.	Samani 2023	37057233	Control group not exclusively consisted of women without breast malignancy

40.	Fagundes 2012	22004465	Serological EBV antibody detection – no molecular detection of EBV genome
41.	Fagundes 2013	22746260	Serological EBV antibody detection – no molecular detection of EBV genome and lack of control group
42.	Hu 2016	27333046	Molecular detection of EBV genome only in breast cancer patients – lack of control group
43.	Fina 2001	11259092	Dependent samples- control samples consisted of histologically normal breast tissue collected from the peripheral margins of malignant breast tumors
44.	Labrecque 1995	7805038	Molecular detection of EBV genome only in breast cancer patients – lack of control group
45.	Fuentes-Pananá 2016	26910355	Dependent samples- control samples consisted of histologically normal breast tissue collected from the peripheral margins of malignant breast tumors
46.	Liao 2017	28940489	Other type of molecular analysis-no molecular detection of EBV genome
47.	Savu 2008	28940489	Molecular detection of EBV genome only in breast cancer patients – lack of control group
48.	Chu 2001	11485915	Molecular detection of EBV genome only in breast cancer patients – lack of control group
49.	Chu 1998	9500191	Molecular detection of EBV genome only in breast cancer patients – lack of control group
50.	El-Shinawi 2016	26508152	Dependent samples- control samples consisted of histologically normal breast tissue collected from the peripheral margins of malignant breast tumors
51.	Preciado 2005	15737034	Control group not exclusively consisted of women without breast malignancy (women with precancerous breast lesions were included)

52.	Deshpande 2002	12218080	Molecular detection of EBV genome only in breast cancer patients – lack of control group
53.	Shukla 2018	29382466	EBV molecular detection in different biological materials (lymph node tissue)
54.	Thorne 2005	15714061	Molecular detection of EBV genome only in breast cancer patients – lack of control group
55.	Hachana 2011	22024152	Dependent samples- control samples consisted of histologically normal breast tissue collected from the peripheral margins of malignant breast tumors
56.	Antonsson 2012	22916092	Dependent samples- control samples consisted of histologically normal breast tissue collected from the peripheral margins of malignant breast tumors
57.	Kleer 2022	12118114	Study population not meeting inclusion criteria
58.	Yahia 2018	29336321	Molecular detection of EBV genome only in breast cancer patients – lack of control group
59.	Herrmann 2003	12559053	Molecular detection of EBV genome only in breast cancer patients – lack of control group
60.	Trabelsi 2008	18227005	Molecular detection of EBV genome only in breast cancer patients – lack of control group
61.	Bau 2007	17494053	EBV molecular detection in different biological materials (blood)
62.	Gumus 2006	17310842	Dependent samples- control samples consisted of histologically normal breast tissue collected from the peripheral margins of malignant breast tumors
63.	Tsai 2007	16865407	Molecular detection of other viral genomes, no EBV or BLV genome detection
64.	Tsai 2005	15602723	Control group not exclusively consisted of women without breast malignancy (samples of thyroid tumors were included)

65.	Angeloni 2001	11287458	Serological EBV antibody detection – no molecular detection of EBV genome and lack of control group
66.	Chen 2000	11051224	EBV molecular detection in different biological materials (serum) and no molecular detection of EBV genome
67.	De Oliveira 2022	35901341	Molecular detection of EBV genome only in breast cancer patients – lack of control group and use of other detection method apart from PCR
68.	Metwally 2021	34188499	EBV molecular detection in different biological materials (blood) in the control group
69.	Ghaffari 2021	33935050	Molecular detection of EBV genome only in breast cancer patients – lack of control group
70.	Jiang 2018	29485413	Dependent samples- control samples consisted of histologically normal breast tissue collected from the peripheral margins of malignant breast tumors
71.	Zhang 2016	26729199	EBV molecular detection in different biological materials (blood) and no molecular detection of EBV genome
72.	Reza 2015	26514536	Dependent samples- control samples consisted of histologically normal breast tissue collected from the peripheral margins of malignant breast tumors
73.	Khabaz 2013	24049539	Dependent samples- control samples consisted of histologically normal breast tissue collected from the peripheral margins of malignant breast tumors
74.	He 2012	22095765	Serological EBV antibody detection – no molecular detection of EBV genome
75.	Kadivar 2011	21207256	Control group not exclusively consisted of women without breast malignancy (patients with precancerous breast lesions were included)
76.	Aguayo 2011	21699721	Molecular detection of EBV genome only in breast cancer patients – lack of control

			group
77.	Hussein 2013		EBV molecular detection with other methods apart from PCR
78.	Peng 2014	25088618	EBV molecular detection with other methods apart from PCR (mass spectrometry was the primary detection method)
79.	Torfi 2021		Dependent samples- control samples consisted of histologically normal breast tissue collected from the peripheral margins of malignant breast tumors
80.	Barzegar 2021	34345390	BLV molecular detection in different biological materials (bovine milk)
81.	Yamanaka 2022	35585539	Molecular detection of BLV genome only in breast cancer patients – lack of control group
82.	Buehring 2019	30940091	BLV molecular detection in different biological materials (blood) and lack of control group
83.	Buehring 2014	24750974	Other type of molecular analysis (in vitro cell line model)
84.	Khalilian 2019	31252065	BLV molecular detection in different biological materials (serum) in the control group
85.	Adekanmbi 2021	34653545	BLV molecular detection in different biological materials (blood)
86.	Corredor-Figueroa 2021	34064361	Study population not meeting inclusion criteria
87.	Zhang 2016	27724949	BLV molecular detection in different biological materials (blood)
88.	Olaya-Galán 2022	35359744	Other type of molecular analysis (in vitro cell line model)
89.	Saito 2020	31776677	Molecular detection of BLV genome only in breast cancer patients – lack of control group
90.	Amato 2023	37517027	Dependent samples- control samples consisted of histologically normal breast tissue collected from the peripheral margins of malignant breast tumors

91.	Canova 2021	33997236	Case group not exclusively consisted of breast malignancy (patients with benign breast diseases were included)
92.	Lorenzetti 2010	21042577	Case group not exclusively consisted of breast malignancy (patients with precancerous breast lesions were included)
93.	Khan 2022	35264739	Case group not exclusively consisted of breast malignancy (patients with benign breast diseases were included)
